# Comparative finite element analyses of stress exerted on the dentin by intraradicular posts

**DOI:** 10.1590/0103-644020246083

**Published:** 2024-12-16

**Authors:** Erica Feleti Lorençon Dettogne, Kênia Maria Pereira Soares de Toubes, Luis Fernando dos Santos Alves Morgan, Paulo Isaías Seraidarian, Lucas Moreira Maia, Frank Ferreira Silveira

**Affiliations:** 1 Department of Dentistry, Pontifical Catholic University of Minas Gerais, Belo Horizonte, MG, Brazil; 2 UNIUBE - University of Uberaba, Uberaba, MG, Brazil; 3 Department of Restorative Dentistry, Federal University of Minas Gerais, Belo Horizonte, MG, Brazil; 4 Adjunct Professor, Endodontic Especialization Course, Itaúna University, MG, Brazil

**Keywords:** Finite element analysis, Dental stress analysis, Computer-aided design, CAD-CAM, Root canal restorative materials, Intraradicular post technique, Nonvital tooth

## Abstract

The amount of residual dentin thickness and tooth position in the dental arch is crucial to determine whether an intraradicular post should be used. This study aimed to compare stress distribution on the root dentin of an endodontically treated tooth rehabilitated with CAD/CAM milled glass fiber posts (MP), cast metal posts (CMP), or prefabricated posts (PP) with or without ferrule support, using the finite element method. Materials and methods: A human upper central incisor was selected, scanned, and treated endodontically. The canal was then prepared for post-placement and scanned again for the fabrication of digital posts. The geometries of MP and CMP were based on the measurements made by digital scanning of the root canal. In contrast, the geometry of PP was determined by technical drawings provided by the manufacturer, taking into account the post-space preparation and the cement. Six digital models were established: MP1 with ferrule support, MP2 without ferrule support; CMP1 with ferrule support, CMP2 without ferrule support; PP1 with ferrule support, and PP2 without ferrule support. The simulation was performed using the finite element method. Results: Oblique forces were identified as more relevant to the metallic element compared to vertical forces. The presence of a ferrule was considered a protective factor for the remaining tooth structure. When the ferrule was absent, stresses were more evenly distributed in MP compared to CMP and PP. Posts with a higher modulus of elasticity were associated with higher and unequal stresses in the root dentin, which could predispose the tooth to fractures. Conclusions: As for the restoration set as a whole, it can be concluded that stress distribution on the root dentin was more evenly distributed on the milled post than on the other posts when a ferrule was absent, and the oblique forces were more detrimental to the tooth structure than vertical forces and that ferrule support was a protective factor for the remaining tooth structure. Clinical significance: The results of the study provide valuable information on how different types of intraradicular posts and the presence of a ferrule affect the stress distribution in dentin. In the absence of a ferrule, milled and customized computer-aided design/computer-aided manufacturing (CAD/CAM) glass fiber posts can exhibit better fracture resistance and adhesion strength than prefabricated fiberglass posts and metal posts.

## Resumo

Objetivo: a quantidade de dentina remanescente e a posição do dente na arcada dentária são cruciais para determinar se um pino intrarradicular deve ser utilizado. O objetivo deste estudo foi comparar a distribuição de tensões na dentina radicular de um dente tratado endodonticamente reabilitado com pino de fibra de vidro fresado CAD/CAM (MP), pino de metal fundido (CMP) ou pino pré-fabricado (PP) com ou sem suporte de férula, por meio do método dos elementos finitos. Materiais e métodos: um incisivo central superior humano foi selecionado, escaneado e tratado endodonticamente. O canal foi então preparado para colocação de pinos e digitalizado novamente para a fabricação de pinos digitais. As geometrias dos MP e CMP foram baseadas nas medidas feitas pelo escaneamento digital do canal radicular, enquanto a geometria dos PP foi determinada por desenhos técnicos fornecidos pelo fabricante, levando em consideração o preparo do espaço do pino para o cimento. Foram estabelecidos seis modelos digitais: MP1 com suporte de férula, MP2 sem suporte de férula; CMP1 com suporte de férula, CMP2 sem suporte de férula; PP1 com suporte de férula e PP2 sem suporte de férula. A simulação foi realizada pelo método dos elementos finitos. Resultados: As forças oblíquas foram identificadas como mais relevantes para o elemento metálico em comparação com as forças verticais. A presença de férula foi considerada fator de proteção para a estrutura dentária remanescente. Quando a férula estava ausente, as tensões foram distribuídas de maneira mais uniforme em MP em comparação com CMP e PP. Pinos com maior módulo de elasticidade foram associados a tensões maiores e desiguais na dentina radicular, o que poderia predispor o dente a fraturas. Conclusões: quanto ao conjunto de restauração como um todo, pode-se concluir que a distribuição de tensões na dentina radicular foi distribuída de maneira mais uniforme no pino fresado do que nos outros pinos quando a férula estava totalmente ausente, e as forças oblíquas foram mais prejudiciais a estrutura dentária do que as forças verticais e que a presença da férula foi um fator de proteção para a estrutura dentária remanescente.

## Introduction

Loss of tooth structures, such as proximal surfaces in anterior teeth, marginal ridge, and enamel bridges in posterior teeth, as well as pulp chamber roof, reduce resistance, as such losses accumulate, making the weakest teeth more fracture-prone ^1^ ^,^ ^2^. In this scenario, the placement of intraradicular posts is often necessary to reshape and restore the function of a weakened tooth. These posts provide retention of the restoration set ^2^ ^,^ ^3^.

Posts are the main mechanisms whereby mechanical forces are transferred to the root, and they can interfere with stress distribution and consequently lead to different failure modes (e.g., cohesive failure in the post and the core, adhesive failure, or root fracture). Therefore, evaluating the biomechanical behavior of different intraradicular post systems and their implications on the remaining root is crucial for the clinical decision about the composition of the post that should be used ^4^ ^,^ ^5^ ^,^ ^6^.

Scientific evidence indicates that the material used for the fabrication of intraradicular posts has a direct impact on their performance. In other words, variations in the modulus of elasticity of different materials affect the mechanical behavior of the posts in the root canal. The higher the modulus of elasticity, the smaller the capacity of the post to distribute the masticatory forces evenly, thus increasing the stress on the dentin. Stiffer materials transfer the stress through the restored tooth directly to the remaining tooth structure, causing stress concentration, thus increasing the risk of fracture ^4^ ^,^ ^6^. The literature has shown greater longevity for teeth restored with prefabricated glass fiber posts than for those teeth restored with metal posts. This can be explained by the fact that glass fiber posts have a modulus of elasticity similar to that of dentin ^4^ ^,^ ^6^ ^,^ ^7^.

Nevertheless, quite recently, CAD/CAM glass fiber posts have been developed as a feasible option for the restoration of endodontically treated teeth in which a sizable amount of tooth structure was lost. MPs fit better onto the root canal walls, with greater retention using attrition force on, and reduction of, the cement line, causing the volume of cement to decrease and gaps to form at the adhesive interface ^8^, resulting in a unique bloc system of intraradicular retention ^4^ ^,^ ^6^. Processing techniques and characteristics of the fiber such as composition, type of fiber, fiber-matrix relationship, arrangement of the fiber in the matrix, shape, and diameter have a direct influence on the mechanical performance of posts ^4^ ^,^ ^6^ ^,^ ^9^. However, the literature on this restoration technique still appears to lack consistency.

The presence of a ferrule is another clinically relevant factor in the case of endodontically treated teeth with major coronal structure loss. The literature largely describes the importance of a minimally adequate dentin height for the reduction of intraradicular stress and fractures ^10^ ^,^ ^11^ ^,^ ^12^ ^,^ ^13^. The searched databases, however, did not reveal any comparative study of CAD/CAM posts in the presence or absence of ferrule assessed by the 3D finite element method ^14^. This method reliably simulates the natural oral environment, avoids destructive experimentation, provides good reproducibility, yields accurate results, and is time-saving ^15^.

Accordingly, this study aimed to compare stresses on the root dentin of an upper central incisor subjected to vertical and oblique masticatory forces rehabilitated with MP, CMP, or PP posts in the presence or absence of ferrule using the finite element method.

## Materials and Methods

The present study was approved by the Research Ethics Committee of the university, process no. 5.594.716. The study followed the guidelines established by the Brazilian National Health Council and complied with Resolution 196/96.

### Sample selection

Ten human upper central incisors were initially selected. The teeth were donated by the tooth bank of the university.

The eligibility criteria used included clinical criteria such as similar size, absence of anatomical variation, and absence of root dilaceration; radiographic criteria including absence of root canals with atresia, abruptly curved canals, internal root resorption, and incomplete root formation; and tomographic criteria like regular internal anatomy of the root canal and similar thickness of dentinal walls on all surfaces. The exclusion criteria were teeth with narrow canals, abrupt curvatures, internal root resorption and incomplete rhizogenesis.

One tooth was then selected for analysis. The selected tooth exhibited root canal centering ability with similarly thick dentinal walls on the buccal, lingual, and proximal surfaces, without anatomical variation, and with a wide root canal. Subsequently, this tooth was scanned to obtain a prototype upper central incisor using a CAD machine (Primescan, Dentsply Sirona, Bensheim, Germany)

### Preprocessing

After access cavity preparation, the canal was prepared with ProTaper Next (Dentsply Sirona, Ballaigues, Switzerland) using the X1, X2, X3, and X4 sequence up to the working length. The tooth was filled with gutta-percha cones and AH Plus Jet cement (Dentsply, Konstanz, Germany).

### Intraradicular post-space preparation

After endodontic treatment, the coronal area of the specimen was removed. An Exacto preparation bur #5 (Angelus, Londrina, PR, Brazil) specifically tailored to root canals was employed for intraradicular post placement. The bur was previously selected according to the canal diameter and attached to a low-speed contra-angle handpiece, according to the manufacturer’s instructions. A 13-mm space was drilled and 4 mm of gutta-percha was maintained at the apical third.

### Scanning

The CAD phase was done using an intraoral scanner (Primescan, Dentsply Sirona, Bensheim, H, Germany) to scan the intraradicular post space and to obtain the MP and CMP digital models.

### Single-unit crown preparation

The geometric parameters suggested by the lithium disilicate glass-ceramic manufacturer were followed for full-crown preparation: 1 mm for buccal, proximal, and palatal surfaces, chamfer margin, and round gingival-axial angles.

**Figure 1:**
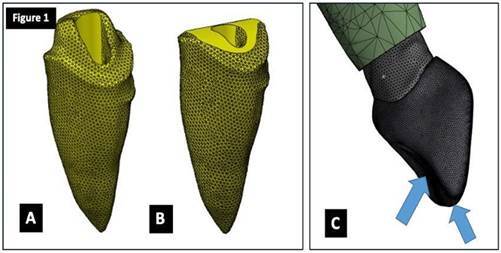
(A) Root with ferrule; (B) Root without ferrule; (C) Direction of masticatory force applied to the models.

Cavity preparation with a 2-mm ferrule was performed above the preparation finish line on the mesial, distal, and buccal surfaces and with a 1-mm ferrule on the palatal surface (Figure 1A).

For unferruled models, the remaining tooth structure was removed using the SolidWorks Corps software (Dassault Systemes, Waltham, MA, USA) at the height of the crown preparation finish line, keeping the chamfer margin (Figure 1B).

### Reverse engineering

The PP consisted of an Exacto glass fiber post (Angelus, Londrina, PR, Brazil) #0.5 built directly in the SolidWorks Corps software (Dassault Systemes, Waltham, MA, USA) based on the technical drawing provided by the manufacturer, resulting in a 200-µm cement line. The increase in volume of the cement line might have occurred because of the larger wear caused by the bur, considering that the extent of abrasion depends on the operator’s experience.

The digital construction of the MP and CMP models was based on the scanning of this space by the CAD scanner (Primescan, Dentsply Sirona, Bensheim, Hessen, Germany). The scanned images were exported to DentalCad (Exocad GmbH, Darmstadt, Hesse, Germany) to obtain the geometry of the post. A 50-µm space was used for the removal of the retention area, and this measurement was set as the cement line for both materials.

Thereafter, the measurements for the external geometry and full-crown fitting were obtained.

All of the images generated by DentalCad (Exocad GmbH, Darmstadt, Hesse, Germany) were exported to Solid Works, where the point cloud was changed into solid elements for the assembly of the structures.

### Experimental design

Three different intraradicular systems were used, organized into six experimental groups (Box 1).

**Box 1:**
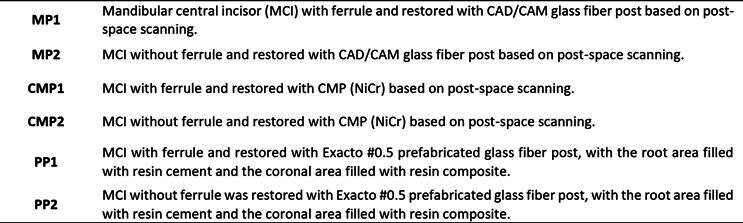
Description of the experimental groups

### Processing (SEM simulation)

After the preprocessing phase, the models were exported to SolidWorks for SEM simulation using Ansys Workbench 19R3 (Ansys Inc., Canonsburg, Pennsylvania, USA) and subjected to numerical processing through the finite element mesh with Ansys supplemental proceedings.

To simulate the biomechanical behavior of each model and the mechanical properties of tooth structures, supporting structures, and restorative materials, the values described in the literature were used to facilitate the comparison of results (Tables 1, 2, and 3).

**Table 1:** Mechanical properties of tooth structures, supporting structures, and restorative materials.

Material	Young’s modulus (GPa)	Poisson coefficient	Source
Dentin	18.6	0.31	Borcic et al. (2005)
Periodontal ligament	0.0118	0.45	Ribeiro (2004)
Cortical bone	13.7	0.3	Ko et al. (1992)
Cancellous bone	1.37	0.3	Ko et al. (1992)
Gutta-percha	0.00069	0.45	Ko et al. (1992)
Resin cement (Rely X Arc-3M ESPE, MN, USA)	5.5	0.24	Campos et al. (2011)
Resin composite (Bis-Core, Bisco, Schaumburg, USA)	12	0.33	Campos et al. (2011)
Zinc phosphate cement	13.7	0.33	Campos et al. (2011)
Lithium disilicate glass ceramic (IPS Empress 2)	103	0.24	Campos et al. (2011)
Chrome-cobalt alloy (cast metal post)	218	0.33	Campos et al. (2011)
Exacto® glass fiber post	30-40	0.22	Provided by the manufacturer
Fiber Cad®	20	0.3	Provided by the manufacturer

**Table 2:** Glass fiber mechanical properties

	Fiber Cad®	Exacto®
Flexural strength*	1,100 Mpa	1,000 to 1,200 Mpa
Mean fiber diameter*	33 μm	12.1 μm

*source: provided by manufacturer /Fiber Cad® (Angelus, Londrina, PR, Brazil) / Exacto® (Angelus, Londrina, PR, Brazil).

**Table 3:** Glass fiber post-composition

Composition (w%)	Glass fiber	Epoxy resin	
Exacto®*	75-80%	20-25%	Provided by the manufacturer
Fiber Cad®*	75-80%	20-25%	Provided by the manufacturer

*source: provided by manufacturer /Fiber Cad® (Angelus, Londrina, PR, Brazil) / Exacto® (Angelus, Londrina, PR, Brazil).

### Masticatory force

Two loading protocols were used to simulate the application of masticatory force (Figure 1C). The first protocol consisted of an oblique load of 100 N applied at 133 degrees from the long axis of the tooth towards the region between the middle third and the incisal surface of the palatal concavity of tooth 11, which corresponds to the contact area of tooth 41. The second protocol consisted of a vertical load of 100 N applied parallel to the long axis of the tooth towards the region that includes the incisal surface of tooth 11, which corresponds to the contact area of tooth 41.

## Results

### Quantitative analysis

When the force vector was applied vertically, the maximum tensile stress was low, but greater in the MP2 and PP2 groups, on the palatal surface of the root in all groups. The sites of application varied as follows: MP and PP1 at the middle third of the root on the palatal surface; MP2 and CMP2 on the palatal surface at the canal entrance; CMP1 on the palatal surface in the ferrule area; PP2 on the palatal surface at the canal entrance.

Compressive stresses with a vertical load were greater in the MP2 and PP2 groups. The compressive stress occurred on the buccal surface of the root in all groups, as follows: MP1, CMP1, and PP1 at the middle third of the root on the buccal surface, and MP2, CMP2, and PP2 on the buccal surface at the preparation finish line (Table 4).

**Table 4:** Maximum tensile and compressive stresses subjected to a vertical load.

Model	Tensile stress (MPa)	Location	Compressive stress (MPa)	Location
MP1	10.88	The middle third of the root on the palatal surface	-40.0	The middle third of the root is on the buccal surface.
MP2	36.32	The palatal surface at the canal entrance	-192.8	The buccal surface at the preparation finish line
CMP1	10.0	Palatal surface, ferrule area	-24.50	The middle third of the root is on the buccal surface.
CMP2	25.49	Palatal surface, canal entrance	-60.0	The buccal surface at the preparation finish line
PP1	10.71	The middle third of the root on the palatal surface	-36.21	The middle third of the root is on the buccal surface.
PP2	48.28	Palatal surface canal entrance	-140.71	The buccal surface at the preparation finish line.

When the force vector was applied obliquely, maximum tensile stress was high in all groups, with higher values for the MP2 and PP2 groups, on the palatal surface of the root, as follows: MP1, CMP1, and PP1 in the ferrule area and MP2, CMP2, and PP2 on the palatal surface at the canal entrance. Compressive stresses with an oblique load were higher in the MP2 and PP2 groups, on the buccal surface of the root, as follows: MP1, CMP1, and PP1 at the middle third of the root and MP2, CMP2, and PP2 on the buccal surface at the preparation finish line (Table 5).

**Table 5:** Maximum tensile and compressive stresses subjected to an oblique load.

Model	Tensile stress (MPa)	Location	Compressive stress (MPa)	Location
MP1	139.38	Ferrule area palatal surface	-92.894	The middle third of the root on the buccal surface
MP2	512.65	Palatal surface Canal entrance	-531.01	Preparation finish line on the buccal surface
CMP1	136.94	Ferrule area palatal surface	-92.543	The middle third of the root on the buccal surface
CMP2	373.04	Palatal surface canal entrance, and middle third of the root	-338.66	Preparation finish line on the buccal surface
PP1	144,3	Ferrule area palatal surface	-90,472	The middle third of the root on the buccal surface
PP2	646,67	Palatal surface canal entrance	-611,81	Preparation finish line on the buccal surface

### Qualitative analysis

The findings demonstrate that oblique forces were more detrimental to the tooth element than vertical forces. The main locations of compressive and tensile stress concentration for vertical and oblique forces are described below.

### Vertical forces

**Figure 2:**
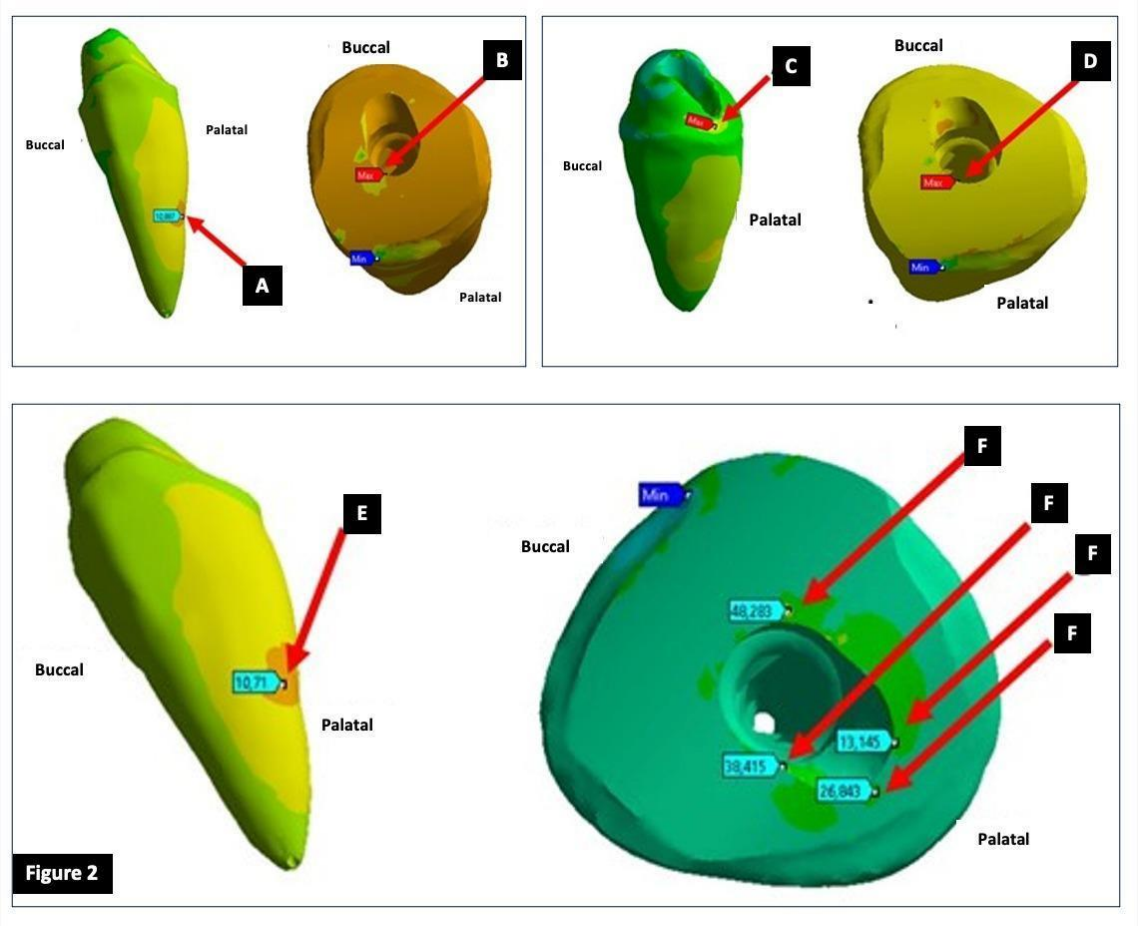
(A) Location of tensile and compressive stresses on MP1; (B) Location of tensile and compressive stresses on MP2; (C) Location of tensile and compressive stresses on CMP1; (D) Location of tensile and compressive stresses on CMP2; (E) Location of tensile and compressive stresses on PP1; (F) Location of tensile and compressive stresses on PP2.

The maximum tensile stress on MP1 was concentrated at the middle third of the root on the palatal surface (Figure 2 A), whereas that of MP2 (Figure 2 B) was concentrated on the palatal surface at the canal entrance. The CMP1 (Figure 2 C) occurred on the palatal surface in the ferrule area, whereas that of CMP2 was concentrated on the palatal surface at the canal entrance (Figure 2 D). Finally, maximum tensile stress on PP1 was concentrated in the middle third on the palatal surface (Figure 2 E), whereas that of PP2 was concentrated on the palatal surface at the canal entrance (Figure 2 F).

### Oblique forces

**Figure 3. (A,:**
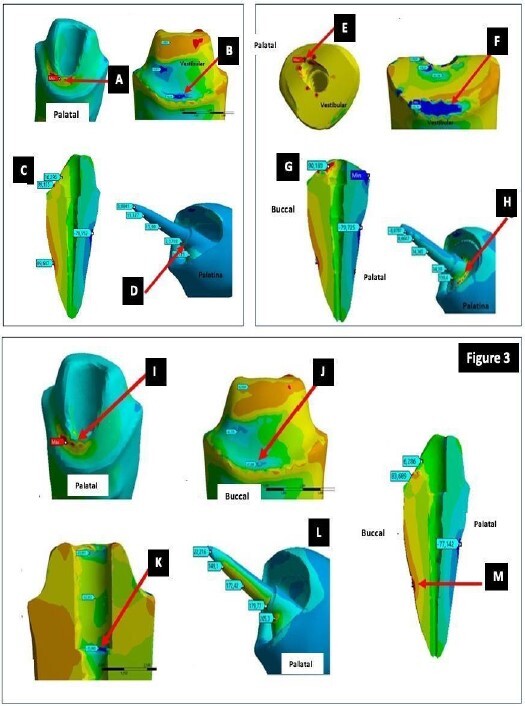
B, C, D) Location of tensile and compressive stresses on MP1 and location of stress on the post; (E, F, G, H) Location of tensile and compressive stresses on MP2 and location of stress on the post; (I, J, K, L, M, N) Location of tensile and compressive stresses on CMP1 and location of stress on the post

In milled posts groups the maximum tensile and compressive stresses on MP1 were concentrated in the ferrule area on the palatal surface (Figure 3 A). On the buccal surface, corresponding to the internal angle and preparation finish line (Figure 3 B), there was a concentration of compressive stress, and peak compressive stress was observed in the region. The inner ferrule area showed evenly distributed stress. Stress was evenly distributed along the root, with concentration at the middle third, but low stress was observed. At the cervical third, on the palatal surface of the post, there was peak tensile stress, which decreased along the post (Figure 3 C, D). MP2 occurred on the palatal surface in the inner area of the canal (Figure 3 E). On the buccal surface corresponding to the preparation finish line (Figure 3 F), there were areas of concentrated and high compressive stress and peak compressive stress occurred in that region. Areas of high stress were also observed at the canal entrance (Figure 3 G). In the inner area of the canal, there was a higher concentration of stress at the cervical third. Stress was evenly distributed along the root, and concentrated at the middle third of the root, but stress was low in that region. There was peak tensile stress at the cervical third on the palatal surface of the post (Figure 3 H).

**Figure 4:**
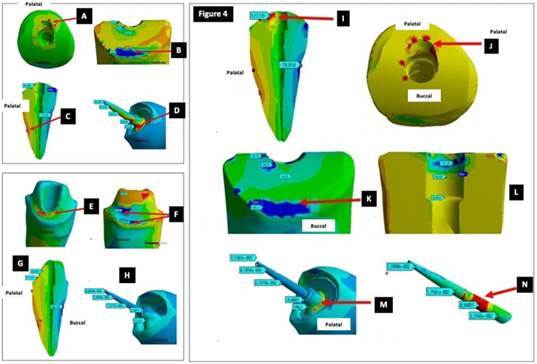
(A, B, C, D) Location of tensile and compressive stresses on CMP2 and location of stress on the post; (E, F, G, H) Location of tensile and compressive stresses on PP1 and location of stress on the post; (I, J, K, L, M, N) Location of tensile and compressive stresses on PP2 and location of stress on the post.

For cast metal posts groups, maximum tensile stress on CMP1 was concentrated in the cervical region on the palatal surface at the canal entrance (Figure 3 I). Maximum compressive stress was observed at the preparation finish line, on the buccal surface (Figure 3 J). Stress was concentrated at the cervical third of the inner area of the canal preparation (Figure 3 K). There was a predominance of tensile stress on the palatal surface along the entire post (Figure 3 L). High stress was observed at the middle third of the root on the palatal surface (Figure 3 M). The maximum tensile stress on CMP2 occurred in the cervical region on the palatal surface and at the middle third of the root on the palatal surface (Figure 4 A). Maximum compressive stress in this model was observed at the crown preparation finish line on the buccal surface of the root (Figure 4 B). High tensile stress was concentrated at the middle third of the root (Figure 4 C). Peak tensile stress was observed at the cervical third on the palatal surface of the post. These forces are transferred to the middle and apical thirds, with high tensile stress (Figure 4 D).

Finally, in prefabricated post groups, compressive and tensile stresses on PP1 were very similar to those observed for the MP. Maximum tensile stress in this model was concentrated on the palatal surface in the inner ferrule area (Figure 4 E) and maximum compressive stress occurred on the buccal surface at the internal angle and preparation finish line (Figure 4 F). These forces were transferred to the middle and apical thirds, but very low stress was observed (Figure 4 G). Tensile stress was concentrated at the cervical third of the post (Figure 4 H). For PP2, this model was concentrated on the palatal surface at the canal entrance (Figure 4 I, J). Maximum compressive stress occurred on the buccal surface at the preparation finish line, indicating that the peak compressive stress was observed in that region (Figure 4 K, L). Stress was more evenly distributed on the inner area of the canal preparation, with tensile stress concentration on the buccal surface at the top of the preparation (Figure 4 M). Tensile stress was concentrated on the cervical third of the post and there was little transfer of stress along the post (Figure 4 N).

## Discussion

According to the maximum stress criterion (Rankine criterion) used, the applied tensile stress and oblique force ^16^ were the most detrimental to the root dentin, and for that reason, these findings will be discussed in further detail.

In the presence of ferrule, CMP showed less stress on the buccal surface of the dentin, at the height of the inner ferrule area, indicating that most of the stress was transferred directly from the crown to the post and the root dentin from the internal preparation finish line, with stress concentration on the root. However, peak stress can occur randomly at some points**,** leading to uneven stress, which can eventually result in catastrophic fractures ^17^. Previous studies have concluded that the materials used for the fabrication of intraradicular posts, with a higher modulus of elasticity, can cause high and uneven stress on the root dentin and predispose tooth fracture, as observed in the present study ^17^ ^,^ ^18^ ^,^ ^19^ ^,^ ^20^ ^,^ ^21^.

The stress produced by the different models could be attributed to the superior adaptation of the MP to the canal, which resulted in a reduction in the thickness of the cement line. It is important to highlight that thicker cement lines can cause deformations at the interface during occlusion, compromising the stability and integrity of the union between the post and the tooth structure, in addition to affecting marginal retention, which increases the risk of long-term failure ^18^.

In the present study, the PM post used zinc phosphate cement, with a simulated cementation line of 50μm, while the PP and MP posts were cemented with Rely X resin cement, with thicknesses of 200μm and 50μm, respectively. Post customization led to a decrease in resin cement thickness, which not only minimized void formation, but also increased tensile strength, reinforcing a more robust bond for the MP post. These results corroborate the findings of previous studies.Bellan et al. ^19^ ^,^ ^20^ ^,^ ^21^.

Void formation is also a concern, as thicker layers of resin cement can create points of weakness that impair the bond ^19^ ^,^ ^22^. Furthermore, the choice of the type of resin cement is crucial, since cements that require adhesive application tend to generate higher pull-out forces compared to self-adhesive cements. Therefore, paying attention to the thickness and type of cement is essential to guarantee ideal bond strength and the durability of long-lasting treatments ^18^ ^,^ ^19^ ^,^ ^20^.

The fracture analysis, with a comparison between models with and without ferrule, showed some similarity between the groups, but the fracture was more severe in the models without ferrule. This finding is in line with those of previous studies ^(^ ^12^ ^,^ ^13^ ^,^ ^23^ ^,^ ^24^ ^,^ ^25^ demonstrating that maintaining approximately 2 mm of the tooth structure above the preparation finish line or the gingival margin is advantageous. In the present study, the ferrule on the palatal surface measured 1.0 mm because of its removal during endodontic access, which is clinically frequent.

Ferrule was an important protective factor for the remaining tooth structure. In all models**,** the absence of ferrule significantly increased the stress on the dentin at the cervical third, especially in the prefabricated glass fiber model, which exhibited higher stress, followed by the MP and CMP, respectively. The presence of ferrule did not change the site where stress occurred, but it significantly reduced the intensity of stress, thereby minimizing the chances of irreparable fractures. These findings concur with those reported in other studies ^1^ ^,^ ^24^ ^,^ ^25^ **,** demonstrating that teeth prepared with ferrule are less prone to catastrophic fractures.

When an unferruled model was simulated, the stress in the cervical region of the dentin on MP was less critical than on PP. This can be explained by the larger diameter of the fibers found in MP, which leads to greater flexure of the post and a smaller cement line, resulting in evenly distributed stress (26-28). When compared to the CMP model, however, stress was higher in PP in the cervical region, possibly due to the high modulus of elasticity of CMP, reducing the tooth’s tendency to bend and making the coronal structure more rigid by transferring the bending point to the more apical area. This can predispose to catastrophic fractures ^29^ ^,^ ^30^ ^,^ ^31^ ^,^ ^32^.

In all models, peak stress on the post occurred in the cervical region and the stress was distributed along the post, showing that PP was more likely to bend than MP. This may be explained by the fiber architecture and by the linear correlation between volume and flexural strength shown in a previous study ^33^, suggesting that a larger volume of fibers may provide the post with higher fracture resistance. Fiber diameter should also be taken into account as studies have demonstrated its correlation with flexural strength ^6^ ^,^ ^34^. The MP assessed in this study has a larger mean fiber diameter, according to the manufacturer, and that may explain the higher stress on the remaining dentin in this group when compared to PP. However, higher flexural strength is an advantage of MPs over PPs in that excessive deformation of the post can lead to cement fracture and loss of retention, affecting the longevity of the restoration ^18^ ^,^ ^21^ ^,^ ^26^ ^,^ ^27^ ^,^ ^28^.

This study is subject to limitations, such as the static nature of the applied force, which cannot accurately reproduce the complexity of masticatory forces in the real oral cavity. Additionally, some simplifications were used in the characterization of materials for finite element studies, which may not fully reflect reality. Even though the present study simulated an analysis of the behavior of the remaining tooth structure using mathematical models, it likely made a contribution by introducing some improvements when compared to previous studies. Among these improvements, the use of a method that simulates complex geometry with high resolution stands out, allowing the detailed reproduction of the internal anatomy of the canal, including its peculiarities and irregularities.

Furthermore, the study also focused on simulating and standardizing the fitting of the intraradicular post in a manner closely aligned with what is observed in clinical practice. , The clinical relevance of the study is significant as it provides valuable insights that can enhance restorative practices. By selecting appropriate post systems and ensuring proper ferrule design, dentists can improve the longevity of endodontically treated teeth and reduce the likelihood of complications. It is important, however, to assess the efficiency of this new post through more robust prospective clinical trials.

## Conclusion

Within the limitations of this study, we can state that Ferrule's models demonstrated a more uniform stress distribution, which contributes to reducing the risk of tooth fractures. It was observed that posts with a higher modulus of elasticity generated higher and unequal tensions in the root dentin, increasing the predisposition to fractures. Furthermore, the analysis revealed that, in the absence of ferrule, the stress distribution in the root dentin was more balanced in MP models compared to the other models. These results indicate that MP represents a viable and effective alternative as intraradicular posts for the restoration of weakened teeth with total ferrule loss, from a biomechanical standpoint.
